# Couverture vaccinale et facteurs associés à la vaccination des professionnels de santé contre la COVID-19 dans la ville de Yaoundé en juillet 2022

**DOI:** 10.11604/pamj.2025.50.97.42581

**Published:** 2025-04-09

**Authors:** Séverine Olga Blanche Koïngakiawa, Joel Lonla Nzougouat, Brice Essomba Edzoa, Calvin Tonga

**Affiliations:** 1 Ecole des Sciences de Santé, Université Catholique d’Afrique Centrale, Yaoundé, Cameroun,; 2 Institut de Formation et de Recherche Démographique, Yaoundé, Cameroun,; 3 Délégation Régionale de la Santé Publique du Centre, Yaoundé, Cameroun,; 4 Programme Elargi de Vaccination, Ministère de la Santé Publique, Yaoundé, Cameroun

**Keywords:** COVID-19, vaccination, facteurs associés, professionnels de santé, COVID-19, vaccination, associated factors, healthcare professionals

## Abstract

**Introduction:**

la vaccination est l´un des moyens efficaces de lutte contre la COVID-19. Les professionnels de santé comptent parmi les cibles prioritaires du fait de leur forte exposition aux risques infectieux. Au regard de couverture vaccinale nationale globalement faible, nous nous sommes intéressés au statut vaccinal des professionnels de santé ainsi qu´aux facteurs associés. L´objectif général a été de déterminer la couverture vaccinale et les facteurs associés à la vaccination contre la COVID-19 chez les professionnels de santé de Yaoundé.

**Méthodes:**

nous avons effectué une étude transversale, quantitative, basée sur une enquête menée auprès de 621 participants issus de 125 formations sanitaires, réparties en 3 strates, incluant tous les districts de santé de Yaoundé du 06/06 au 09/07/2022. La taille de l´échantillon a été calculée suivant l´approche recommandée par l´Organisation Mondiale de la Santé (OMS) (2015) pour l´estimation de couverture vaccinale par la méthode d´échantillonnage en grappe, à deux degrés, avec 10% de précision, pour une couverture vaccinale anticipée à 25% et un intervalle de confiance à 95%. Les analyses bivariées et multivariées ont permis d´identifier les associations.

**Résultats:**

la couverture vaccinale des professionnels de santé de Yaoundé a été estimée à 42,0% (IC 95%: 38,2% - 46,0%) dont 1,3% de couverture vaccinale vérifiée. Les facteurs socioprofessionnels (catégorie de la formation sanitaire (FOSA), secteur d´activité et unité de soins) et psychosociaux (expérience de la COVID-19 et perception du vaccin contre la COVID-19) sont statistiquement associés au statut vaccinal des professionnels de santé.

**Conclusion:**

la couverture vaccinale chez les professionnels de santé de Yaoundé reste faible, ces résultats présentent des éléments pertinents d´évaluation et d´ajustement des stratégies en vue de l´amélioration de la couverture vaccinale des professionnels de santé tant au niveau régional que national.

## Abstract (en)

**Introduction:**

vaccination is one of the most effective COVID-19 control strategies. Because of their high exposure to infectious risks, healthcare workers are considered as priority targets. Given the overall low vaccination coverage in the country, we investigated the vaccination status of healthcare workers and the associated factors. The general objective was to determine vaccination coverage and factors associated with COVID-19 vaccination among healthcare workers in Yaoundé.

**Methods:**

we conducted a quantitative, cross-sectional study based on the survey of 621 participants from 125 health facilities, divided into 3 strata, including all the health districts in the city of Yaoundé, from 06/06 to 09/07/2022. The sample size was calculated following the WHO recommended approach for estimating vaccine coverage using the two-stage cluster sampling method, with 10% precision, for an anticipated vaccine coverage of 25% and a 95% confidence interval. Bivariate and multivariate analyses were used to identify associations.

**Results:**

vaccination coverage among healthcare workers in Yaoundé was estimated at 42.0% (95% CI: 38.2% - 46.0%), including 1.3% verified vaccination status. Socio-professional (category of health facility, activity sector and care unit) and psychosocial (level of experience of COVID-19 and perception of the COVID-19 vaccine) factors are statistically associated with vaccination.

**Conclusion:**

vaccination coverage among health professionals in Yaoundé remains low, these results provide relevant information for evaluating and adjusting strategies to improve vaccination coverage among healthcare workers at both regional and national levels.

## Introduction

La pandémie de la COVID-19 a entrainé des pertes en vies humaines et impacté tous les secteurs de la vie sociale [1-12]. À l´épreuve de cette crise, le système de santé mondial a réagi rapidement, déployant divers moyens dont le recours à la vaccination [13-16]. La vaccination est l'un des moyens sûrs et efficaces de lutte contre les infections, y compris la COVID-19 [17]. L´OMS recommande une couverture vaccinale de 80 à 90%, pour atteindre l´immunité collective permettant d´interrompre la circulation du virus et éviter sa mutation vers des formes plus pathogènes [18]. Les gouvernements ont fixé des objectifs nationaux de couverture vaccinale qui permettraient d´assurer le contrôle de la pandémie. Au Cameroun, l´objectif était de 70%, les professionnels de santé étant une cible prioritaire [19].

Bien que la COVID-19 ne soit plus une urgence de santé publique de portée internationale, la vaccination contre la COVID-19 reste recommandée [20,21]. Plutôt que les nourrissons et femmes enceintes habituellement ciblés, cette vaccination concerne les adultes aux premiers rangs desquels les professionnels de santé. La nouveauté du vaccin expose à des défis dont l´hésitation vaccinale et la désinformation [19].

Les couvertures vaccinales basées sur les données administratives sont souvent peu fiables, notamment dans les pays en développement, du fait de la faible précision du dénominateur, des défauts d´enregistrement ou de compilation des actes vaccinaux, et de l´altération des données par les acteurs de la chaîne [22]. D´où le recours aux enquêtes qui lorsqu´elles sont bien réalisées génèrent des résultats fiables, permettant une évaluation objective des efforts de vaccination et de la qualité des données administratives, et servant de base pertinente de décision [23]. À notre connaissance, aucune estimation de la couverture vaccinale contre la COVID-19 chez les professionnels de santé n´a été menée au Cameroun. Notre étude visait à déterminer la couverture vaccinale et les facteurs associés à la vaccination contre la COVID-19 chez les professionnels de santé de la ville de Yaoundé.

Les résultats de ce travail pourraient servir à l´évaluation et l´amélioration de la performance et des stratégies de vaccination. Ils enrichiraient la compréhension de l´attitude des personnels de santé vis-à-vis de la vaccination contre la COVID-19 et pourraient orienter les stratégies de vaccination contre les formes mutées du coronavirus, les infections émergentes et ré-émergentes à caractère épidémique, et l´introduction des nouveaux vaccins.

## Méthodes

**Figure 1:**
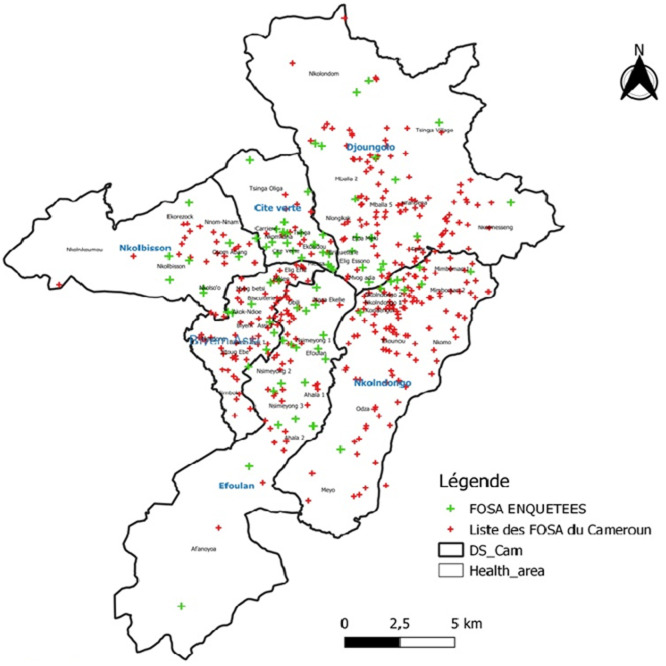
cartographie des sites d'enquête

**Cadre de l´étude:** cette étude a été menée dans tous les districts de santé (DS) de la ville de Yaoundé (Figure 1), chef-lieu de la région du Centre, capitale politique et ville la plus peuplée du Cameroun. Située dans le département du Mfoundi et couvrant une superficie de 304 Km^2^, la ville est bornée au nord-ouest par le département de la Lékié, au sud-ouest par le département de la Mefou-et-Akono, au sud par le département de la Mefou-et-Akono, au nord par l´arrondissement d´Okola, et à l´est par le département de la Mefou-et-Afamba [24]. Elle compte le plus grand nombre de personnels de santé. C´est dans cette ville qu´a été déclaré le premier cas de COVID-19 au Cameroun, le 06 mars 2020. La ville comptait alors 6 DS et 60 aires de santé (AS). Au mois de mars 2022, la ville comptait 901 formations sanitaires (FOSA) répertoriées dans le DHIS2.

**Type d´étude:** cette étude est quantitative, de type transversal, à visée descriptive et analytique.

**Participants à l´étude:** ils étaient constitués de tous les professionnels de santé exerçant dans les FOSA de la ville de Yaoundé. Ont été inclus dans l´étude tout professionnel de santé exerçant dans les FOSA de la ville de Yaoundé pendant la période d´enquête et ayant accepté de participer à l´étude.

**Conception de l´étude:** l´étude a été menée en 3 strates. La strate 1 regroupait les FOSA de catégories 1 et 2 (hôpitaux généraux et centraux); la strate 2 était constituée des FOSA de la catégorie 3 et 4 (hôpitaux régionaux et hôpitaux de district); et la strate 3 des FOSA des catégories 5 et 6 (centres médicaux d´arrondissements et centres de santé intégrés). La taille de l´échantillon a été déterminée par strate suivant la méthode de sondage en grappe recommandée par l´OMS [22], en utilisant la formule T=B*C*D*E; la taille minimale d´échantillon attendue était de 126, 130 et 357 respectivement pour chacune des strates soit un total de 613 participants (Tableau 1). Toutes les FOSA des strates 1 et 2 ont été incluses dans l´étude. Le nombre de participants par FOSA a été calculé en divisant la taille d´échantillon par le nombre de FOSA de la strate. La sélection des participants a été faite par tirage aléatoire à partir de la liste des professionnels de santé de la FOSA. La sélection des FOSA dans la strate 3 a été faite par double tirage aléatoire: un premier pour sélectionner les AS, et un autre basé sur la liste des FOSA actives dans l´AS. Lorsque l´effectif au sein d´une FOSA était inférieur à 7, une FOSA de la même AS était sélectionnée suivant la même démarche pour compléter le nombre de répondants.

**Tableau 1:** paramètres à prendre en compte pour le calcul de la taille de l’échantillon

	Paramètres	Strate 1	Strate 2	Strate 3	TOTAL
B	Taille effective de l’échantillon	95	95	95	
C	Effet de conception	1	1	3	
D	Nombre de FOSA à visiter pour trouver un répondant	1	1	1	
E	Facteur de correction de la non-réponse	1,25	1,25	1,25	
n	Nombre de FOSA incluses	9	13	51	73
t	A enquêter par FOSA	14	10	7	
Taille d’échantillon		**126**	**130**	**357**	**613**

* Niveau de précision fixé à 10%, Intervalle de Confiance de 95%, Couverture vaccinale anticipée à 25%, Taux de non-réponse anticipé de 20%. FOSA: Formation sanitaire

**Collecte des données:** elle a été menée du 06 juin au 09 juillet 2022, soit 3 mois après la quatrième campagne d´intensification de la vaccination contre la COVID-19. Les données ont été collectées à l´aide de deux questionnaires sur papier, prétestés dans deux FOSA du DS de la Cité Verte, qui n´ont pas été incluses dans l´enquête. Les insuffisances identifiées au cours du prétest ont permis d´améliorer les versions finales. Le premier questionnaire a servi à collecter les données sociodémographiques, socioéconomiques, socioprofessionnelles, socioculturelles, et psychosociales qui ont servi de variables indépendantes, ainsi que le statut vaccinal des participants. Le second a servi à collecter les données relatives aux formations sanitaires retenues pour l´enquête, notamment le nombre total de personnels par catégorie professionnelle, en vue de la pondération des données. L´information a été préalablement donnée aux participants sur la nature, l´objectif et l´intérêt de l´étude, et leur consentement éclairé a été sollicité.

**Considérations éthiques:** cette étude a obtenu la clairance éthique du Comité d´Ethique Institutionnel de la Recherche pour la Santé Humaine (CEIRSH) de l´Ecole des Sciences de la Santé de l´Université Catholique d´Afrique Centrale, sous le numéro 2022/0202190/CEIRSH/ESS/MSP. Les autorisations d´enquêtes ont été obtenues des responsables des FOSA de catégorie 1, 2 et 4. Pour les FOSA de catégories 5 et 6, l´autorisation d´enquête a été obtenue de la délégation régionale de la Santé Publique pour le Centre et des lettres d´introduction ont été obtenues des chefs de DS. Les questionnaires étaient tous anonymes. Aucune contrepartie financière n´a été requise ou offerte aux répondants. Seuls les participants éligibles, ayant préalablement donné leur consentement éclairé par écrit ont participé à l´étude.

**Traitement et analyse des données:** ils ont été faits à l´aide des logiciels CsPro, SPSS et Stata, le seuil de significativité était de 5% pour un intervalle de confiance à 95%. Afin de faciliter les analyses, certaines variables ont été recodées. De même, pour des raisons de synthèse, des indices synthétiques ont été construits pour résumer certains facteurs. Le traitement des données manquantes a été fait en utilisant la valeur moyenne pour les variables quantitatives, et le mode pour les variables qualitatives. Les analyses bivariées et multivariées ont été menées afin d´identifier les associations statistiquement significatives. Pour contrôler les facteurs confondants, un test du VIF a été utilisé.

## Résultats

L´enquête a été menée auprès de 621 professionnels de la santé en exercice dans 125 des 901 FOSA de la ville de Yaoundé, représentant les 3 strates de l´étude.

### Description des participants

La population d´étude est à prédominance féminine (72,5%), avec une moyenne d´âge de 33 ans +/-0.37 et un niveau d´étude supérieur à BAC+3. Les participants ayant un indice de niveau de vie faible sont plus représentés (45,0%). La majorité des participants de l´étude (52,5%) travaillent dans le secteur public. Quant à la catégorie professionnelle des participants, les infirmiers(ères) sont majoritaires (32,1%) dans toutes les strates. Les participants proviennent pour l´essentiel des aires géographiques forestière (48,4%) et Grassfield (34,6%); ils sont majoritairement chrétiens (86,1%) et d´expression française (71,0%); (Tableau 1).

Au niveau psychosocial, les participants sont classés en grande partie dans le groupe d´indice de connaissance et de perception moyen (48,8%). Cette distribution est observée dans toutes les strates, exceptée la strate 3 où ils sont principalement du groupe d´indice de connaissance et de perception élevés (43,7%). La majorité des participants (38,1%) ont un indice moyen d´expérience personnelle de la COVID-19 dans leur entourage, excepté dans la strate 1 où l´expérience de la COVID-19 chez les participants est beaucoup plus élevée (51,1%). Les participants ont une perception favorable du vaccin contre la COVID-19 à 40,2%. Cette tendance s´observe également à l´intérieur des strates (33,8%, 40% et 47,5%; Tableau 2).

**Tableau 2:** caractéristiques des participants

Modalité		Ensemble (n=621)	Strate 1 (n=133)	Strate 2 (n=130)	Strate 3 (n=158)
**Caractéristiques sociodémographiques**					
Sexe (n=621)	Masculin	27,5%	31,6%	26,2%	26,5%
	Féminin	72,5%	68,4%	73,8%	73,5%
Non-réponse,(%)		0,0%	0,0%	0,0%	0,0%
Âge (n=596)	Moyenne [écart-type]	33,0 [0,37]	38,8 [0,32]	34,8 [0,29]	32,7 [0,27]
	20-35 ans	55,9%	36,6%	55,7%	67,5%
	35-50 ans	35,4%	51,1%	40,2%	30,1%
	Plus de 50 ans	4,7%	12,2%	4,1%	2,3%
Non-réponse,(%)		4,0%	1,5%	6,2%	4,5%
Statut matrimonial (n=621)	Seul	38,9%	42,9%	40,0%	37,2%
	En couple	61,1%	57,1%	60,0%	62,8%
Non-réponse,(%)		0,0%	0,0%	0,0%	0,0%
Niveau d'étude (n=621)	Secondaire	32,9%	23,3%	26,9%	38,5%
	Baccalauréat + 3	27,5%	36,8%	40,0%	40,5%
	Supérieur à Baccalauréat +3	39,6%	39,8%	33,1%	20,9%
Non-réponse,(%)		0,0%	0,0%	0,0%	0,0%
**Caractéristiques socio-économique**					
Statut de résidence (n=621)	Maison familiale/Locataire	76,3%	61,0%	76,8%	81,8%
	Propriétaire	23,7%	39,0%	23,2%	18,2%
Non-réponse,(%)		0,0%	0,0%	0,0%	0,0%
Moyen de déplacement	Marche à pied /transport en commun	75,5%	68,4%	70,3%	80,0%
	Véhicule personnel	24,5%	31,7%	29,7%	20,0%
Non-réponse,(%)		0,0%	0,0%	0,0%	0,0%
Possibilité d'effectuer un ou plusieurs voyages par avion (n=621)	Pas du tout/Rarement	89,5%	85,6%	85,4%	92,4%
	Quelques fois au moins	30,5%	39,9%	38,3%	24,3%
Niveau de revenu (n=611)	Faible	46,8%	41,5%	37,5%	51,9%
	Moyen	52,2%	58,5%	62,5%	46,3%
	Elevé	0,9%	0,0%	0,0%	1,5%
	Ne souhaite pas répondre	0,2%	0,0%	0,0%	0,4%
Non-réponse,(%)		0,2%	0,1%	0,0%	0,0%
**Caractéristiques socioprofessionnelle**					
Secteur d'activité (n=612)	Public	52,5%	98,6%	62,5%	31,6%
	Privé laïc	35,7%	1,4%	0,0%	61,5%
	Privé confessionnel	10,6%	0,0%	37,5%	5,0%
Non-réponse,(%)		1,4%	0,0%	0,0%	2,5%
Nombre d'années de service (n=609)	Moins de 5ans	40,1%	26,6%	41,9%	44,5%
	5-10 ans	31,4%	23,8%	30,8%	34,5%
	Plus de 10 ans	27,3%	49,6%	27,3%	18,9%
Non-réponse,(%)		1,9%	3,1%	1,5%	2,8%
Catégorie professionnel (n=606)	Médecin/Pharmacien(ne)	17,0%	28,8%	22,1%	11,2%
	Infirmier	32,1%	33,0%	27,7%	33,5%
	Sage-femme/Maïeuticien	6,6%	6,6%	9,0%	5,6%
	TMS	12,5%	8,0%	13,7%	13,8%
	Aide-soignant(e)	23,7%	17,4%	20,9%	27,2%
	ATMS	7,7%	6,1%	6,7%	8,7%
Non-réponse,(%)		2,4%	0%	0%	4,2%
Unité de soins (n=592)	Groupe 1a	39,7%	33,1%	44,2%	40,7%
	Groupe 2b	39,0%	50,3%	34,8%	36,2%
	Groupe 3c	21,2%	16,6%	21,0%	23,1%
Non-réponse,(%)		4,2%	0,8%	3,8%	5,7%
**Caractéristiques socioculturelles**					
Religion (n=600)	Christianisme	86,1%	84,9%	91,3%	84,6%
	Islam	4,9%	8,9%	2,3%	4,3%
	Ne souhaite pas répondre	9,0%	6,2%	6,5%	11,1%
Non-réponse,(%)		3,4%	0,8%	0,8%	5,3%
Région d'origine (n=620)	Sahélienne	5,5%	8,9%	5,2%	4,5%
	Forestière	48,4%	45,7%	48,8%	49,2%
	Grassfield	34,6%	35,2%	35,9%	34,0%
	Côtière	9,1%	10,1%	7,3%	9,3%
	Ne souhaite pas répondre	2,4%	0,1%	2,9%	3,1%
Non-réponse,(%)		0,2%	0,01%	0,0%	0,0%
Langues parlées (n=613)	Français (F)	71,0%	71,6%	76,7%	68,6%
	Anglais (A)	2,2%	1,5%	1,3%	2,8%
	Bilingue (F/A)	26,8%	26,9%	22,0%	28,6%
Non-réponse,(%)		1,3%	0,2%	1,2%	1,8%

a) Accueil, Tri, Consultations externes et urgences; b) Laboratoire, Pharmacie et banque de sang; c) Prise en charge hospitalière, Soins intensifs/Réanimation et bloc opératoire

### Estimation de la couverture vaccinale

**Figure 2:**
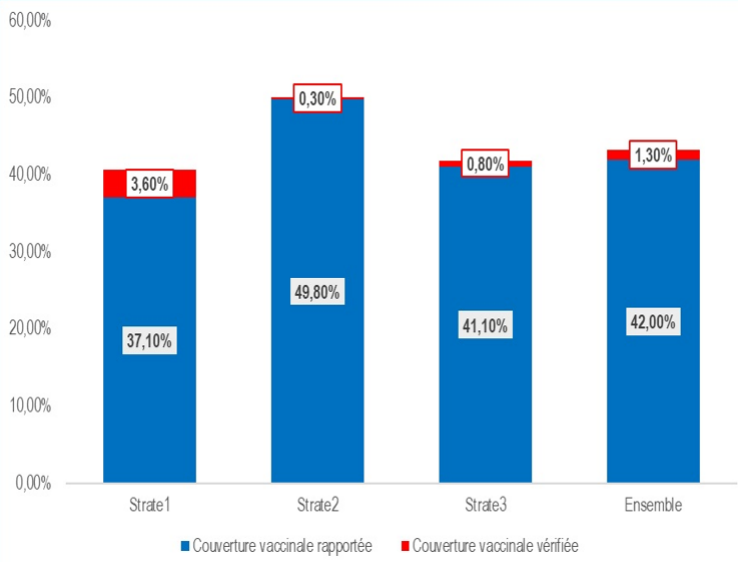
taux de couverture vaccinale par strate et dans l´ensemble

La couverture vaccinale est estimée à 42% (IC 95%: 38,2% - 46,0%), similaire à celle basée sur les déclarations des participants regroupant la couverture vaccinale apportée et la couverture vaccinale vérifiée. Désagrégée par strate, elle est de 37,1% (IC 95%: 29,3% - 45,7%) pour la première, 49,8% (IC95%: 41,2% - 58,3%) pour la seconde et 41,1% (IC 95%: 36,1% - 46,3%) pour la troisième (Figure 2). La couverture vaccinale vérifiée (présentation de la carte de vaccination) est de 1,3%; elle est de 3,6% dans la strate 1, et inférieure à 1% pour les autres strates.

### Facteurs associés à la couverture vaccinale

**Caractéristiques sociodémographiques:** le personnel féminin est plus souvent vacciné que le masculin (couvertures vaccinales de 44,6% et 35,3% respectivement). L´on observe par ailleurs une proportion plus élevée (50,5%) de personnes vaccinées parmi les personnels de la tranche de 35 - 50 ans comparé à ceux des tranches 20 - 35 ans et plus de 50 ans. La proportion de vaccinés parmi le personnel vivant en couple est plus élevée que chez ceux qui vivent seuls (44,7% et 37,7% respectivement). Par ailleurs, la proportion des personnes vaccinées ayant un niveau supérieur à BAC+3 (46,7%) ou un niveau BAC+3 (45,0%) est plus importante que celle des participants ayant un niveau secondaire (36,4%; Tableau 3). Cependant, les différences de couvertures vaccinales ainsi observées ne sont pas statistiquement significatives.

**Tableau 3:** analyses bivariées et multivariées

Caractéristiques		Statut vaccinal			Analyse bivariée		Analyse multivariée		
		Vaccinés	Non vaccinés	Total					
		n (%)	n (%)	N (%)	OR	P-Value	aOR	95% [IC]	P-Value
		261 (42,0%)	360 (58,0%)	621 (100%)					
Sociodémographiques									
Sexe	Féminin	200 (44,6%)	249 (55,4%)	449 (100%)	Réf.	0,545	Réf.	Réf.	Réf.
	Masculin	61 (35,3%)	111 (64,7%)	172 (100%)	0,75		1,132	0,602-2,127	0,696
Âge	20-35 ans	120 (34,5%)	228 (65,5%)	348 (100%)	Réf.	0,054	Réf.	Réf.	Réf.
	35-50 ans	108 (50,5%)	106 (49,5%)	214 (100%)	1,802		1,078	0,507-2,295	0,842
	Plus de 50 ans	13 (46,4%%)	15(53,6%%)	28 (100%)	1,839		1,683	0,37-7,659	0,496
Statut matrimonial	En couple	171 (44,7%)	221 (60,7%)	382 (100%)	Réf.	0,634	Réf.	Réf.	Réf.
	Seul	90 (37,7%)	149 (62,6%)	239 (100%)	0,766		1,005	0,66-1,531	0,981
Niveau d'étude	BAC+3	92 (45,0%)	111 (55,0%)	205 (100%)	Réf.	0,134	Réf.	Réf.	Réf.
	Secondaire	90 (36,4%)	157 (63,6%)	247 (100%)	1,295		1,697	0,93-3,09	0,083
	Supérieur à BAC+3	79 (46,7%)	90 (53,4%)	169 (100%)	1,523		1,05	0,47-2,37	0,898
Socioéconomiques									
Niveau de vie	Faible	87 (30,8%)	196 (69,2%)	284 (100%)	Réf.		Réf.	Réf.	Réf.
	Moyen	70 (46,5%)	80 (53,5%)	150 (100%)	1,74	0,001*	1,184	0,65-2,15	0,578
	Elevé	103 (55,5%)	83 (44,5%)	187 (100%)	2,42		1,62	0,96-2,73	0,07
Socioprofessionnelles									
Strate	Strate 1	49 (37,2%)	84 (62,8%)	133 (100%)	0,81	0,146	0,16	0,06-0,41	0,000*
	Strate 2	64 (49,8%)	66 (50,2%)	130 (100%)	1,42		0,36	0,11-1,11	0,089
	Strate 3	147 (41,1%)	211(58,9%)	358(100%)	Réf.		Réf.	Réf.	Réf.
Secteur d'activité	Public	178 (54,5%)	149 (45,5%)	326 (100%)	Réf.	0,001*	Réf.	Réf.	Réf.
	Privé laïc	57 (26,4%)	158 (73,6%)	214 (100%)	0,301		0,12	0,05-0,31	0,000*
	Privé confessionnel	23 (34,9%)	43 (65,1%)	66 (100%)	0,452		0,3	0,10-0,92	0,036*
Nombre d'années d'expérience	Moins de 5 ans	78 (30,9%)	159 (69,1%)	237 (100%)	Réf.	0,001*	Réf.	Réf.	Réf.
	5-10 ans	77 (37,8%)	116 (62,2%)	193 (100%)	1,287		1,078	0,51-2,29	0,842
	Plus de 10 ans	94 (52,0%)	70 (48,0%)	164 (100%)	2,521		1,683	0,37-7,65	0,496
Catégorie professionnelle	Personnels médicaux et Pharmacien(ne)	53 (50,0%)	53 (50,0%)	106 (100%)	1,736	0,03*	1,025	0,41-2,51	0,957
	Personnels médico-sanitaires	205 (40,8%)	296 (59,2%)	501 (100%)	Réf.		Réf.	Réf.	Réf.
Unité de soins	Groupe 1^a^	88 (42,3%)	120 (57,7%)	208 (100%)	1,076	0,03*	1,245	0,73-2,13	0,421
	Groupe 2^b^	35 (28,4%)	88 (71,6%)	123 (100%)	0,461		0,47	0,25-0,90	0,022*
	Groupe 3^c^	129 (49,4%)	132 (50,6%)	261 (100%)	Réf.		Réf.	Réf.	Réf.
Socioculturelles									
Religion	Christianisme	220 (42,8%)	293 (57,2%)	513 (100%)	Réf.	0,52	Réf.	Réf.	Réf.
	Islam	9 (29,2%)	21 (70,8%)	30 (100%)	0,64		0,4	0,13-1,25	0,112
Aire géographique d'origine	Forestière	131 (42,5%)	177 (57,5%)	377 (100%)	Réf.	0,933	Réf.	Réf.	Réf.
	Grassfield	83 (39,7%)	126 (60,3%)	209 (100%)			1,2	0,64-2,26	0,567
	Côtière	24 (43,8%)	31 (56,2%)	55 (100%)			1,16	0,41-3,27	0,77
	Sahélienne	18 (56,4%)	14 (43,6%)	244 (100%)			1,78	0,70-4,52	0,219
Langue parlée	Français	179 (41,6%)	251 (58,4%)	430 (100%)	1,37	0,08	1,38	0,67-2,83	0,38
	Anglais/Bilingue	80 (44,1%)	102 (55,9%)	182 (100%)	Réf.		Réf.	Réf.	Réf.
Facteurs psychosociaux									
Indice d'expérience de la COVID-19	Faible	45 (26,2%)	128 (73,8%)	173 (100%)	0,38		0,33	0,14-0,79	0,012*
	Moyen	100 (42,7%)	134 (57,3%)	234 (100%)	1,04	0,001*	0,94	0,44-2,00	0,862
	Élevé	116 (54,2%)	98 (45,8%)	213 (100%)	Réf.		Réf.	Réf.	Réf.
Indice de perception sur la COVID-19	Faible	43 (38,1%)	70 (61,2%)	112 (100%)	0,82		1,41	0,65-3,07	0,38
	Moyenne	146 (50,8%)	142 (49,2%)	288 (100%)	1,97	0,003*	0,71	0,35-1,42	0,324
	Élevé	72 (32,6%)	149 (67,4%)	221 (100%)	Réf.		Réf.	Réf.	Réf.
Indice de perception du vaccin contre la COVID-19	Faible	38 (20,7%)	144 (79,3%)	182 (100%)	0,11		0,14	0,08-0,25	0,001*
	Moyenne	54 (29,9%)	126 (70,1%)	180 (100%)	0,26	0,001*	0,2	0,10-0,39	0,001*
	Élevé	169 (69,7%)	73 (30,3%)	243 (100%)	Réf.		Réf.	Réf.	Réf.

**Caractéristiques socioéconomiques:** la proportion de personnes vaccinées est plus élevée (55,5%) parmi les professionnels ayant un niveau de vie élevé. Bien que significative à l´analyse bivariée (p-value=0,001), l´analyse multivariée montre que cette association n´est pas statistiquement vérifiée (p-value = 0.07) (Tableau 3).

**Caractéristiques socioprofessionnelles:** comparée à la strate 1 (37,2%), la proportion de personnes vaccinées est plus élevée dans la strate 2 (49,8%), suivie de la strate 3 (41,1%; OR = 0,16; p-value=0,001). Elle est plus élevée chez les professionnels du secteur public (54,5%; p-value=0,006). Le nombre d´années d´expérience professionnelle semble impacter la vaccination. En effet, les personnes ayant plus de 10 ans d´expérience professionnelle sont plus souvent vaccinées (52%). Aussi, les professionnels médicaux et pharmaciens semblent plus souvent vaccinés, comparés aux médico-sanitaires (50% contre 40,8%). Les professionnels de santé qui travaillent dans les services d´hospitalisation, de soins intensifs et de réanimation sont aussi plus souvent vaccinés que les autres (49,4%; p-value=0,022). En somme, la strate, le secteur d´activité et l´unité de soins sont parmi les facteurs socio-professionnels, ceux qui sont significativement associés au statut vaccinal chez les professionnels de santé (Tableau 3).

**Caractéristiques socioculturelles:** aux analyses bivariées et multivariées, aucune des caractéristiques retenues n´est significativement associée au statut vaccinal (Tableau 3).

**Facteurs psychosociaux:** la proportion de professionnels vaccinés est plus importante chez les professionnels ayant fait l´expérience de la COVID-19 (54,2%). La même tendance est observée chez les personnes ayant un indice de perception élevé du vaccin contre la COVID-19 (69,7%). En ce qui concerne la perception de la maladie, la proportion des personnes vaccinées ayant un indice moyen de la perception de la COVID-19 est nettement supérieure à celle des participants ayant un indice élevé. Toutefois, à l´analyse multivariée, seuls le niveau d´expérience de la COVID-19 (OR=0,33 ; p-value=0,012) et la perception du vaccin contre la COVID-19 sont significativement associés au statut vaccinal (OR=0,14; p-values=0,0001). (Tableau 3). Ainsi, l´adhésion à la vaccination augmente significativement avec l´expérience de la COVID-19 et une perception favorable du vaccin contre la COVID-19.

## Discussion

La vaccination, moyen de prévention et de lutte contre les épidémies et la pandémie, demeure un levier important de la santé publique [17]. La présente étude avait pour objectif de déterminer la couverture vaccinale et les facteurs associés à la vaccination des professionnels de santé contre la COVID-19 dans la ville de Yaoundé. Plus précisément il a été question d´estimer la couverture vaccinale COVD-19 des professionnels de santé exerçant dans les formations sanitaires de la ville de Yaoundé d´une part, et d´identifier les facteurs associés à la vaccination d´autre part, partant de l´hypothèse que les facteurs sociodémographiques, socioéconomiques, socioculturels et psychosociaux influencent le statut vaccinal des professionnels de santé.

**Couverture vaccinale des professionnels de santé:** la couverture vaccinale contre la COVID-19 a été estimée à 42% (IC 95%: 38,2%-46,0%) chez les professionnels de santé de la ville de Yaoundé, indépendamment du schéma vaccinal. Autrement dit, ce résultat inclut toutes les personnes ayant reçu au moins une dose de vaccin contre la COVID-19. La couverture vaccinale vérifiée étant assez faible (1,3%). Cela pourrait s´expliquer par le fait que les participants se déplacent rarement avec leur carte de vaccination (preuve de vaccination), ce qui rend difficile la vérification de leurs déclarations. La réticence à présenter la carte de vaccination pourrait en outre s´expliquer par la crainte d´exposer des informations à caractère personnel. À ce niveau intervient le respect de la liberté des individus auquel nous nous sommes engagés.

Entre les strates l´on observe une disparité significative de la couverture vaccinale. En effet, la strate 2 constituée d´hôpitaux de la 4^e^ catégorie présente une couverture vaccinale plus élevée (49,8%) que celles de la strate 1 (37,2%) et la strate 3 (41,1%). La couverture vaccinale dans la strate 1 mérite une investigation beaucoup plus profonde. En effet, la majorité des hôpitaux (5/9) que constitue cette strate se réclament d´un statut parapublic. Ce qui sous-tend l´aspect d´une grande autonomie similaire à celle des acteurs du secteur privé, ayant leur propre conseil d´administration. Par conséquent, l´on pourrait comprendre la faible couverture vaccinale de la strate 1 à la lumière de l´influence du secteur privé sur le statut vaccinal des professionnels de santé.

Pour revenir à l´estimation de la couverture vaccinale dans son ensemble, nous constatons que cette estimation est comparable à celle publiée par le ministère de la Santé publique au Cameroun: 56,2% pour l´ensemble du pays, 40,7% pour la région du Centre. Pour ce qui est de la ville de Yaoundé, la couverture vaccinale des professionnels de santé est estimée à 48,4% au 30 juin 2022 (il s´agit ici du contact vaccinal) [25]. Cette dernière s´harmonise avec la couverture obtenue à l´issue de notre étude, eu égard à la précision de 10% adoptée parmi les paramètres d´estimation de la cible de notre enquête.

Ayant utilisé l´approche méthodologique d´enquête de couverture vaccinale en grappe selon l´OMS à travers une collecte d´informations directes auprès des professionnels de santé, nous pouvons dire avec assez de certitude que nos résultats traduisent la réalité du terrain. Le faible écart (06,4%) entre les données administratives et les résultats de l´enquête traduit une qualité globalement bonne des données administratives.

**Facteurs associés à la vaccination contre la COVID-19:** l´exploration des facteurs associés à la vaccination a été faite à partir de la théorie du modèle de croyances relative à la santé. Cela nous a permis d´énumérer un certain nombre de facteurs associés à l´adhésion ou non des professionnels de santé à la vaccination contre la COVID-19. Ces facteurs, à la lumière d´études antérieures, ont été contextualisés. L´acceptation du vaccin par les professionnels de santé dépend de plusieurs paramètres que nous appelons facteurs associés, ayant pour but d´influencer la décision d´une personne. Dans cette section, nous présentons les différents facteurs ayant influencé la décision des professionnels de santé de se faire vacciner ou, au contraire, à refuser de le faire. L´objectif a été d´identifier les facteurs qui ont réellement une influence sur le statut vaccinal des professionnels de santé tout en éliminant les facteurs confondants.

Nous n´avons pas trouvé d´association significative entre le genre et le statut vaccinal des professionnels de santé. Cette observation est contraire aux résultats des études de Biswas *et al*. Hajure *et al*. Kumar *et al*. Muhajarine *et al*. et de Navarre *et al*. pour qui le genre était associé à l´adhésion vaccinale. Dans notre contexte, cette absence d´association peut témoigner de la conscience qu´ont les professionnels de santé d´être exposés à la maladie au même titre puisque le risque dans les FOSA ne diffère pas selon qu'on soit homme ou femme. [26-31].

Également, nous avons remarqué que l´âge n´est pas significativement associé au statut vaccinal. Ces observations rejoignent celle de l´étude de Fares *et al*. [32] qui a remarqué que l´âge n´était pas associé à l´adhésion vaccinale chez les professionnels de santé alors que la plupart des revues systématiques de Biswas *et al*. [29] et Luo *et al*. [27], ou des études transversales de El-Sokkary *et al*. [33] et Kumar *et al*. [28] ont montré une association entre l´âge et le statut vaccinal. En effet, les différents résultats de ces études sus-évoquées ont montré que l´adhésion à la vaccination augmentait avec l´âge (? 30 ans), et l´hésitation vaccinale ou le refus augmentait elle aussi avec des âges les moins avancés (> 30 ans) [26-28,33-36]. Bien que ce deuxième cas de figure aurait pu être un facteur significatif chez nous, cela n´a pas été démontré par l´analyse de la régression logistique. Nous pouvons déduire que la variable âge serait un facteur confondant dans notre étude, d´où son élimination après l´analyse multivariée.

Il en va de même pour le niveau d´étude. Il n´est pas significativement associé au statut vaccinal dans notre étude. Alors que Biswas *et al*. [29], dans sa méta-analyse, associait le niveau d´étude élevé (les titulaires d´un PhD) à une disposition favorable à la vaccination chez les professionnels de santé. Cet écart entre ce résultat et le nôtre pourrait s´expliquer par le fait que l´intention vaccinale ne veut pas dire obligatoirement l´acceptation. L´intention est d´ordre abstrait tandis que le fait de se faire vacciner requiert un acte d´engagement concret. En outre, le répondant pourrait donner la réponse qu'il pense satisfaisante pour l'enquête, ce qui ferait que les intentions seraient toujours plus élevées que la vaccination effective. En effet, d´après Aucouturier, il est connu que « les questions hypothétiques (intentions ou anticipations) sont problématiques et peu fiables [37]. Quoi qu´il en soit, nos résultats reflètent la réalité de notre contexte.

L´on pourrait anticiper que le niveau de vie ait une influence sur le statut vaccinal des professionnels de santé comme cela a été démontré au Canada où les analyses ont montré que les personnes de faible niveau de vie adhéraient moins souvent à la vaccination contre la COVID-19 [38]. Fort est de constater que cela n´est pas le cas dans notre étude. En somme, par rapport au statut vaccinal des professionnels de santé de Yaoundé, le niveau de vie serait un facteur confondant.

Par rapport aux professionnels exerçant dans les FOSA des 5^e^ et 6^e^ catégories (strate 3), ceux des 2^e^ et 1^re^ catégories (strate 1) se vaccinent moins souvent. Prenant en compte le fait qu´un nombre non négligeable de vaccinations enregistrées dans les FOSA à Yaoundé sont le fait de la pression hiérarchique (24,4%), nous pourrions dès lors comprendre l´acceptation vaccinale importante du personnel de la strate 3 composée en majorité des infirmiers (33,5%) et des aides-soignants (27,2%). Pourtant Navarre *et al*. [30] dans son étude menée en France, trouvait que « les professions paramédicales » étaient réfractaires au vaccin contre la COVID-19.

S´agissant du secteur d´activité, nous nous sommes rendus à l´évidence qu´il influence significativement le statut vaccinal des professionnels de santé de Yaoundé. En effet, les professionnels exerçant dans le secteur public se vaccinent plus souvent que ceux du secteur privé. Cela rejoint les résultats d´étude de Navarre *et al*. [30] en France et celle d´El-Sokkary *et al*. [33] en Egypte, où il a aussi remarqué que le secteur privé présentait plus de défiance vis-à-vis de la vaccination contre la COVID-19 et donc les professionnels de santé qui s´y trouvent se vaccinent moins souvent. Cette attitude serait-elle liée à un manque de confiance aux autorités sanitaires ou alors à la politique sanitaire en vigueur dans le pays? Nous n´avons pas de réponse à cette question pour le moment. Cela nécessiterait une étude plus spécifique dans ce sens pour mieux cerner ce qui se joue.

L´unité de soins est significativement associée au statut vaccinal. En effet, les professionnels exerçant dans les services des hospitalisations/soins intensifs/réanimations et bloc opératoire se vaccinent 2 fois plus que ceux d´autres services. Cela rejoint les observations faites par Luo *et al*. [27]. Au regard de ces informations et en nous référant à la théorie de la croyance relative à la santé, l´on pourrait se dire que le fait d´être en unité de soins intensifs ou de réanimation met le professionnel face à la réalité des cas graves de la COVID-19 à travers les patients pris en soins, cela les emmène à percevoir la gravité de la COVID-19 et la menace pour leur propre santé, c´est pourquoi ils adoptent le comportement favorable vis-à-vis de la vaccination.

En explorant ces éléments socioculturels, nous cherchions à voir si l´acceptation ou le refus de la vaccination était lié à la culture d´origine des participants. Force est de constater que cela n´a pas été vérifié. Nous concluons donc en disant que le statut vaccinal des professionnels de santé de Yaoundé n´est pas fonction des facteurs socioculturels.

L´expérience de la COVID-19 par les participants ainsi qu´un indice élevé de perception du vaccin contre la COVID-19 augmentent leur adhésion à la vaccination. Cela est en lien étroit avec ce que décrit le modèle de la croyance relative à la santé. Plus la gravité de la menace est perçue ainsi que l´effet bénéfique des mesures de protection, plus l´on adopte un comportement favorable à la santé [39]. C´est pourquoi, dans presque toutes les études menées par d´autres chercheurs, « la perception d´un risque élevé d´exposition au COVID-19 », ou encore la confiance en « la sécurité et l´efficacité des vaccins » sont statistiquement associés à l´adhésion vaccinale chez les professionnels de santé. À l´inverse, « les attitudes négatives ainsi que les mauvaises perceptions » entraînent un refus de se faire vacciner [26,32,33,36,38,40].

### Limites de l´étude

L´étude étant quantitative, elle ne saurait explorer les raisons profondes de l´acceptation de la vaccination ou de son refus chez les professionnels de santé. Une étude qualitative pourrait être envisagée pour explorer davantage les raisons du refus de la vaccination par certains professionnels de santé. Par ailleurs, du fait du caractère ondoyant de l´être humain, il se pourrait que certaines déclarations des participants sur leur statut vaccinal soient biaisées, à cause de la «désirabilité sociale » [41].

## Conclusion

Notre travail sur la couverture vaccinale et les facteurs associés à la vaccination des professionnels de santé contre la COVID-19 dans la ville de Yaoundé a permis d´estimer la couverture vaccinale des professionnels de santé à 42,0%. Cette couverture vaccinale est inférieure à l´objectif de 70% fixé par le pays, mais comparable au résultat administratif qui est de 48,6%, lorsqu´on prend en compte le seuil de précision fixée à 10%. Les facteurs significativement associés à la vaccination sont socioprofessionnels (catégorie de la FOSA, secteur d´activité et unité de soins) et psychosociaux (expérience de la COVID-19 et perception du vaccin contre la COVID-19). Les personnels des services les plus susceptibles de recevoir et de prendre en charge les malades de COVID-19, ceux ayant fait l´expérience de la COVID-19 et ceux ayant un meilleur indice de perception et de connaissance du vaccin se vaccinent plus souvent. La peur des effets indésirables est la principale raison exprimée pour justifier la non-vaccination. Une étude qualitative permettrait d´explorer plus en profondeur ces aspects. Les résultats issus de ce travail confirment la bonne qualité des données administratives et présentent des éléments pertinents d´évaluation et d´ajustement des stratégies en vue de l´amélioration de la couverture vaccinale des professionnels de santé.

### Etat des connaissances sur le sujet

- L´impact de la COVID-19 dans le monde;
- Le développement et la recommandation des vaccins comme instrument de lutte complémentaire aux autres stratégies déjà déployées;
- La réticence à la vaccination contre la COVID-19.

### Contribution de notre étude à la connaissance

- L´étude a estimé de façon précise et fiable la couverture vaccinale des professionnels de santé à 42,0%;
- L´étude montre que les facteurs socioprofessionnels et psychosociaux sont associés à la vaccination des professionnels de santé;
- L´étude a établi un lien entre la catégorie de la formation sanitaire suivant le découpage en vigueur dans le système de santé, le statut vaccinal des professionnels de santé; une bonne connaissance de la maladie et l´expérience individuelle, à travers un proche ou un patient, est associée à une meilleure adhésion à la vaccination.
